# A 15-year review of lightning deaths in Germany—with a focus on pathognomonic findings

**DOI:** 10.1007/s00414-024-03175-6

**Published:** 2024-01-30

**Authors:** Rebecca Bingert, Larissa Bremer, Andreas Büttner, Stefan Nigbur, Ryan Blumenthal, Fred Zack

**Affiliations:** 1 Institute of Legal Medicine, Rostock University Medical Center, Rostock, Germany; 2 Department of Forensic Medicine, University of Pretoria, Pretoria, South Africa

**Keywords:** Pathognomonic findings, Singed hair, Lichtenberg figures, Postmortem examination, Misdiagnosis

## Abstract

Lethal accidents caused by lightning are divided into observed and unobserved events. Pathognomonic or characteristic findings are often overlooked during external postmortem examination. Classical mistakes may be made which may often lead to an incorrect diagnosis. An analysis of 270 fatalities was performed on a historical collection of the Committee for Lightning Conductor Construction for the United Economic Area e. V. (ABBW) on lethal accidents due to lightning that occurred in Germany for the period 1951–1965. Furthermore, a selective literature research was carried out. The aim of the study was to analyze the death scene, the clothing, and the victim. The authors focused on chief findings which were relevant to the correct diagnosis of “death by lightning.” Both Lichtenberg figures and singed body hair were considered pathognomonic for a lightning strike. The question arose as to whether Lichtenberg figures, for example, represented the finding that most often led to the correct diagnosis. Of the 270 lightning-struck victims from the case collection, 129 (47.8%) had singed body hair and 25 (9.3%) had Lichtenberg figures. A comparison of the frequency of the two reported findings, singed body hair versus Lichtenberg figures, has only been performed in the literature for case numbers below 40. This study is the first evaluation of a relatively large number of lethal accidents due to lightning. Singed body hair was reported more frequently in lightning-struck victims than Lichtenberg figures. This study showed that singed body hair is probably more diagnostically important than Lichtenberg figures.

## Background

A lightning strike can cause none, to severe injuries or even death, especially depending on the mode of energy transmission. In the literature, data on the proportion of fatal lightning accidents compared to human injury events diverge widely [1]. In recent decades, there has been an important decrease in fatal lightning accidents in developed nations [2]. In the USA between 1900 and 1925 approximately 3.5 to 7 people per one million people died due to a lightning strike, this rate steadily declined to below 1 after 1960 and even 0.1 per one million in recent years. Similar decreases in fatal lightning strikes per one million people were also found in Canada and in many European countries, including Germany [2, 3].

Fatal accidents caused by lightning are divided into observed and unobserved events. The rarity of the occurrence of fatal lightning accidents, the possibility of events with discrete characteristic findings, lack of knowledge, and insufficient care on the part of the doctor performing the external postmortem examination favor misjudging the correct cause of death [4–10].

The significant decrease in fatal accidents over the past few decades means that the specialist literature, published many years ago, on this unusual type of accident is also of noteworthy importance [10, 11].

One of the aims of the present study was to analyze which findings at the scene and on the victim were pathognomonic or characteristic for the correct diagnosis of “death by lightning.”

A further aim was to determine which injury was reported more frequently in lightning-struck victims: Singed hair or Lichtenberg figures? And whether the Lichtenberg-figure was a finding that more often led to the correct diagnosis.

## Material and methods

An analysis of 412 fatalities was performed on a historical collection of the Committee for Lightning Conductor Construction for the United Economic Area e. V. (ABBW) of cases of accidents caused by lightning strikes with personal injury that occurred in the Federal Republic of Germany between 1951 and 1965 and had previously only been evaluated from an electrotechnical point of view.

The historical collection was handed over to the Institute of Legal Medicine of the Rostock University Medical Center in 2016 by the ABB (Lightning Protection and Lightning Research Committee) of the VDE (Association for electrical engineering, electronics, and information technology) for medical analysis. The collection contained inhomogeneous material as standardized forms, police reports, doctor’s reports, newspaper articles, photos, and hand sketches of the accident’s scene.

On all of the historical material of the case collection, a comprehensive forensic medical review was performed.

Further a selective research of specialist literature on the topic was performed.

## Results

The fatal accidents—included in the study—were evaluated according to the following categories: damaged objects or dead animals on victim’s surroundings, damaged worn clothing/jewelry, characteristic findings on the corpse (Table Table 4 Lightning detection systemsCountry/regionExplanationGermany, BLIDSCommercial lightning detection serviceAustria, ALDISAustrian lightning detection information systemSwitzerlandMeteoSchweizEurope, EUCLIDEuropean Cooperation for Lightning DetectionUSA, NLDNUS National Lightning Detection Network DatabaseCanada, CLDNCanadian Lightning Detection NetworkSouth Africa, SALDNSouth African Lightning Detection Network1).

**Table 1:** Pathognomonic and characteristic findings on victim’s surroundings, worn clothing, jewelry, and the corpse

Victim’s surroundings	Clothing/jewelry	Findings on the corpse
Damaged trees	Mechanical rupture	Signed body hair
Burned grass	Thermic damage	Lichtenberg figures
Dead animals	Metallization	Burned skin
Damaged objects	Lichtenberg figures	Bleeding from the ear canal
		Metallization

### Incidence of accidents caused by lightning strikes with injured persons

Of the 480 collected files examined, 375 fatal accidents were identified as possible and probable lightning strike cases. The collection of cases included 412 dead and 648 injured people. Overall, 237 accidents were exclusively fatal and in 32 incidents, more than one person died.

According to the Federal Statistical Office, 752 deaths due to lightning were recorded for the period from 1951 to 1965 in the Federal Republic of Germany (FRG), so that with 412 deceased from the case collection, a total of 54.8% of the deceased due to lightning were recorded [3] (Table 2).

**Table 2:** Deaths caused by lightning according to the Federal Statistical Office and the case collection from 1951 to 1965

Deaths due to lightning in the Federal Republic of Germany			
Year	Case collection	Federal Statistical Office [3]	Proportion of victims from the case collection among the total number of deaths in the FRG (%)
1951	7	101	6.9
1952	12	48	25.0
1953	34	87	39.1
1954	42	52	80.8
1955	55	91	60.4
1956	59	57	103.5
1957	33	46	71.7
1958	29	33	87.9
1959	30	43	69.8
1960	14	29	48.3
1961	19	44	43.2
1962	13	25	52.0
1963	30	38	78.9
1964	25	35	71.4
1965	10	23	43.5
Total	412	752	54.8

Of the 412 fatal accidents, 382 (92.7%) died directly at the scene and 30 (7.3%) after a latency period that could not be determined in more detail due to missing information in the case collection.

### Characteristic findings on victim’s surroundings

In 235 (57%) of the 412 fatal accidents, information was found on the surroundings of the scene. In 66 (28.1%) of the 235 fatal accidents caused by lightning, it was noted in the case collection that no damage to property or dead animals had been found on the surroundings of the scene. According to this, there were 169 (71.9%) fatal accidents caused by lightning in this collection with information on property damage caused by lightning or dead animals.

**Fig. 1:**
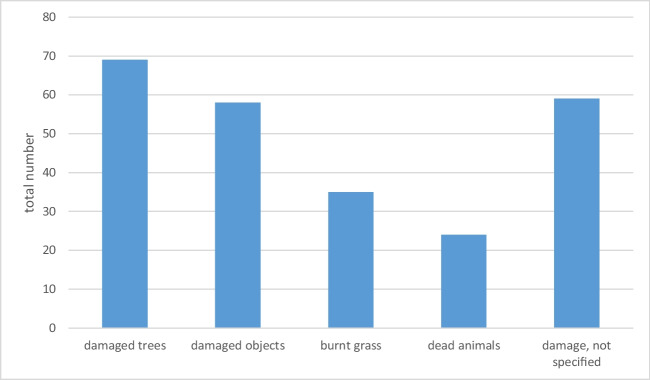
Frequency of characteristic findings in the victim’s environment

With 69 fatal accidents, damage to trees were seen most often, followed by 58 fatal incidents with damage to objects (shovel, rake, etc.), there were 35 fatal accidents with burnt grass and 24 fatal incidents with dead animals, chiefly mammals, in the immediate surroundings of the scene. In 59 fatal accidents, the information was imprecise (damage without naming the object). In no cases were Lichtenberg figures noted other than on the skin of a deceased. Multiple mentions were possible (Fig. 1).

### Characteristic findings on worn clothing/jewelry

**Fig. 2:**
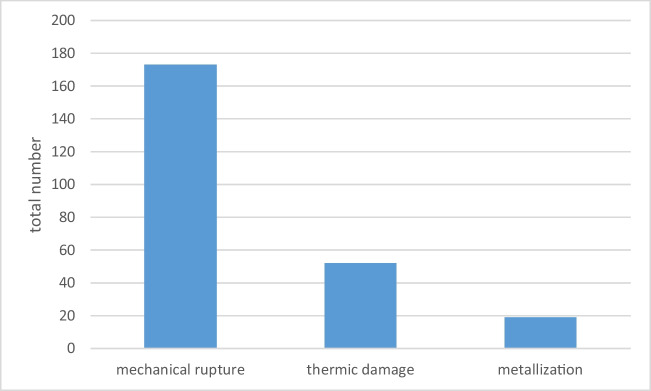
Frequency of characteristic findings on worn clothing/jewelry

Of the 412 fatal accidents, 223 (54.1%) reported findings on the clothing or jewelry worn. Mechanical rupture of the clothing (tearing and tattering) was mentioned with 173 cases, followed by 52 cases of thermic damage and 19 cases had signs suggestive of metallization. Multiple mentions were possible (Fig. 2).

### Pathognomonic and characteristic findings on the corpse

**Fig. 3:**
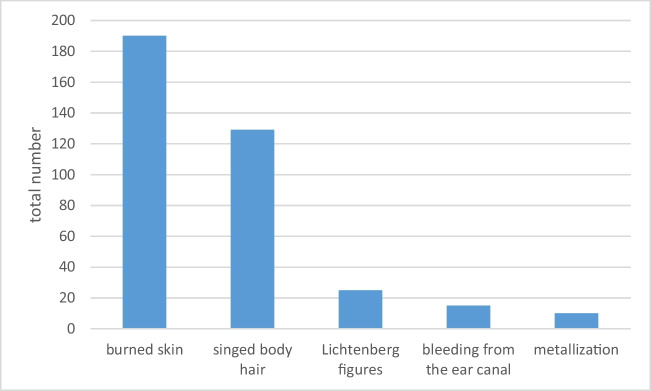
Frequency of characteristic findings on the corpse

Of the 412 fatal accidents, 270 (65.5%) cases contained information about findings on the corpse. Burn injuries were documented in 190 cases, singed body hair in 129 cases, Lichtenberg figures in 25 cases, bleeding from the ear canal in 15 cases, and metallization effects on the skin in 10 cases. Multiple mentions were possible (Fig. 3).

## Discussion

### Difficulties regarding the diagnosis

In particular, the forensic literature shows that numerous problems and misdiagnoses may arise regarding a forensic medical postmortem examination [12]. There are many reasons for an incorrect diagnosis. In many cases, doctors concentrate solely on the corpse, neglecting the immediate surroundings on the scene where the body was found [4, 13].

Another common mistake that can lead to an incorrect diagnosis is to not undress the deceased during an external postmortem examination [6–8]. Another source of error is a lack of diligence of the doctor performing the external postmortem examination, with essential findings being overlooked [4, 6]. Furthermore, the lack of knowledge about pathognomonic or characteristic findings may also lead to an incorrect diagnosis [5–8, 13]. Even after a correctly performed external postmortem examination obtaining the diagnosis correctly can be difficult in cases with discrete or no findings, even after lightning strike [9, 14].

In unobserved accidents, the diagnosis “death by lightning” is made due to the inspection of the surroundings on the scene, the victim’s clothing, including the contents of pockets and jewelry, as well as the findings on the corpse.

For a long time, Lichtenberg figures were regarded as the only pathognomonic finding for the diagnosis “lightning injury.” However, more than 100 years ago, singed body hair of a lightning victim was already considered a very characteristic finding [15].

If an accident due to technical electricity (high voltage) is excluded, both the Lichtenberg figure and singed body hair are considered pathognomonic for a lightning strike. In addition, skin burns and bleeding from the ear canal may sometimes have other causes. There has not yet been a study on the incidence of occurrence of singed body hair after fatal accidents due to lightning in a larger study group.

In a study of 45 deaths from lightning strikes in Florida (USA), Wetli found singed body hair and/or skin burns in 41 (91.1%) of the victims [16]. Unfortunately, no differentiation was made between the two different findings. An analysis of 10 fatal lightning accidents in Australia showed 7 victims with singed body hair, especially head and pubic hair [17]. In a study from Mecklenburg-West Pomerania singed body hair was found in 2 of 4 deceased due to lightning strike [3]. Lifschultz and Donoghue had similar findings in one out of five lightning strikes with fatal consequences [18].

Until the present study, there were no data for larger case numbers of fatal victims due to lightning strike with the occurrence of singed hair.

Of the 270 fatal accidents caused by lightning with information about diagnostic findings, singed hair was documented about five times more frequently than Lichtenberg figures in this study with 129 cases. This result confirms the assumption, based on the small number of cases so far, that after a fatal accident caused by lightning, regarding the pathognomonic findings, singed hair occurs considerably more often than Lichtenberg figures (Table 3).

**Table 3:** Frequency of singed body hair and Lichtenberg figures in the literature

Authors	**Signed body hair**	**Lichtenberg figures**	Total number of cases
Bingert et al.	129	25	270
Blumenthal 2005 [19]	26	0	38
Andrews and Cooper 1992 [17]	7	2	10
Lifschultz and Donoghue 1993 [18]	1	1	5
Schniers 2005 [3]	2	1	4

In addition to the significantly more frequent occurrence, the singed body hair in relation to Lichtenberg figures has two other advantages for the diagnosis. Singed body hair does not disappear after a few hours as a finding and is also easily detectable in victims with darker skin color [19].

On the one hand, skin burns may be caused by other causes, however on the other hand, for example, they can show such characteristic findings due to lightning entry and exit marks that they must be regarded as proof of a lightning strike after a high-voltage accident due to technical electricity has been excluded.

Another finding on an external postmortem examination that refers to the diagnosis “death by lightning” is bleeding from the ear canal following a ruptured tympanic membrane from the barotrauma associated with the lightning strike [20–22]. This injury may occur on one or both sides whereas ruptures of both tympanic membranes are described less frequently. There are different statements in the specialist literature about the frequency of occurrence of tympanic membrane ruptures in relation with an accident caused by lightning. These range from 10% (examination collective with *n* = 5) over 50% (*n* = 66) to over 80% (*n* = 45) [16, 23, 24].

Bleeding from the ear canal in an unobserved accident caused by lightning is rarely sufficient as a diagnostic finding, since there are numerous other causes of bleeding from the ear canal, such as skull base fractures, brain tumors, ear furuncles, or foreign objects. In observed events, on the other hand, in addition to a rupture of the tympanic membrane, a basal skull fracture after a serious fall must also be considered. Cherington et al. reported a case in which bilateral tympanic membrane ruptures combined with thermal damage of a worn jogging shoe were the only findings in an unobserved death from lightning strike [21].

Metallization effects on the skin are relatively rare and have never been the sole finding that led to the diagnosis of “death by lightning” in cases published to date [10, 25].

### Technical support for an accurate diagnosis

Doctors performing the external postmortem examination who are not sure whether a lightning strike caused injuries have been able to get support of regional lightning detection systems for many years. If one is interested, any doctor and any other person can research whether cloud-to-earth lightnings have been registered for an explicit time and location. So far, this service has helped to classify unexplained deaths correctly and to provide scientific research with specific data on the times, locations, and amperages of the detected lightning strikes [22, 24, 26–28]. For example, with the help of the US National Lightning Detection Network (NLDN) (Table 4), it was demonstrated that a cyclist’s acute cardiac arrest, which occurred under cloudless skies, was due to a lightning strike occurring 10 miles (16 km) from the associated thunderstorm cell (“bolt from the blue”) [11].

**Table 4:** Lightning detection systems

Country/region	Explanation
Germany, BLIDS	Commercial lightning detection service
Austria, ALDIS	Austrian lightning detection information system
Switzerland	MeteoSchweiz
Europe, EUCLID	European Cooperation for Lightning Detection
USA, NLDN	US National Lightning Detection Network Database
Canada, CLDN	Canadian Lightning Detection Network
South Africa, SALDN	South African Lightning Detection Network

## Conclusion

Classical mistakes may be made, even by good forensic pathologists, in the forensic examination of lightning-struck victims, and this could be seen as a possible limitation of this study [6].

According to the 15-year retrospective descriptive analysis of the historical case collection on deaths by lightning in Germany, pathognomonic and diagnostic features such as signed body hair, Lichtenberg figures, skin burns, bleeding from the ear canal, and/or metallization [25] effects of the skin were found in more than 95% of the 270 deaths in which information on the results of the external postmortem examination were available.

However, with regard to the frequency of occurrence of the two pathognomonic findings, singed body hair possessed the greatest relevance, since it was reported in almost every death and occurred about five times more frequently than Lichtenberg figures.

In addition to the findings on the corpse, characteristic findings on the worn clothing and jewelry as well as the findings on the scene may also lead to the correct diagnosis of “death by lightning,” in the majority of cases.

## Data Availability

The data sets analyzed for the current study are available upon request from the corresponding author.
